# Case report: hunting the hidden: surgical treatment of chronic silent thrombus in the left ventricle in a young alcoholic patient with myocardial bridging

**DOI:** 10.1186/s13019-023-02414-y

**Published:** 2023-11-09

**Authors:** Fengpu He, Yiping Jiao, Lijun Jiang

**Affiliations:** 1 Department of Cardiovascular Surgery, The First Affiliated Hospital, Zhejiang University School of Medicine, No. 67 Qingchun Road, Hangzhou, 310003 Zhejiang China; 2 Psychological Teaching and Research Department, Non-commissioned Officer Academy of PAP, Hangzhou, China

**Keywords:** Left ventricular thrombus, Systemic embolism, Myocardial bridging, Surgical treatment

## Abstract

**Background:**

A silent left ventricular thrombus is dangerous. The current standard anticoagulation therapy was ineffective in our case or similar, and the outcome was poor.

**Case presentation:**

A 33-year-old man with a silent left ventricular thrombus was detected incidentally by transthoracic echocardiography. After admission, anti-coagulation with low-molecular-weight heparin therapy was carried out. The CAG revealed 70% systolic stenosis in the middle of the right coronary artery along with myocardial bridging. Unfortunately, an acute left temporal embolism emerged 5 days later, then the patient was transferred to the neurology department for further treatment. One month later, the patient underwent left ventricular thrombectomy, ventricular aneurysm resection, and coronary artery bypass grafting (CABG) and was discharged uneventfully after surgery.

**Conclusions:**

Surgical treatment should be a priority for patients with giant or hypermobile left ventricular thrombus or recurrent systemic emboli.

## Background

Left ventricular thrombus (LVT) is uncommonly seen in unselected patients by echocardiogram. It is, however, more commonly found in patients with heart failure and acute myocardial infarction [1]. Silent LVT detected by transthoracic echocardiography (TTE) is rare and dangerous in those with normal cardiac function without any history of cardiac diseases. The current standard treatment strategy for LVT is anticoagulation therapy, including vitamin K antagonist (VKA), direct oral anticoagulants, low molecular heparin, and intravenous unfractionated heparin [2, 3]. Nevertheless, surgical intervention should be considered if systemic embolism emerged [2].

## Case presentation

A 33-year-old man was referred to the cardiac surgery department due to apical space-occupying lesions detected incidentally by TTE during the routine preoperative examination for urinary calculi. In the outpatient department, the patient's blood pressure, heart rate, and percutaneous oxygen saturation were all within normal range, without any signs of acute or chronic cardiac failure. The patient, however, had a more than 10-year history of drinking and smoking.

**Fig. 1:**
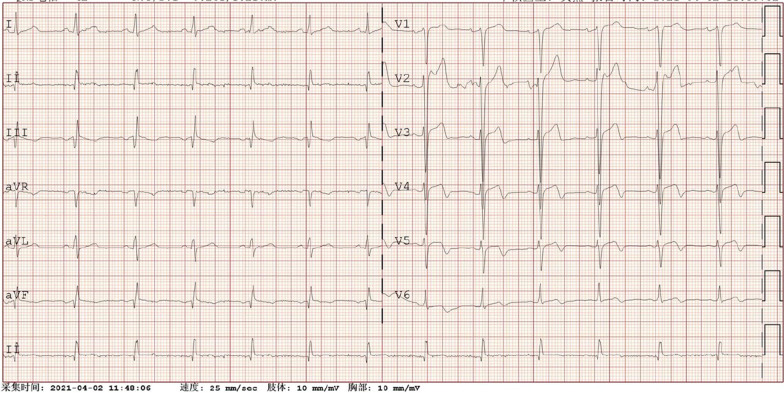
Preoperative electrocardiogram shows sinus rhythm

**Fig. 2:**
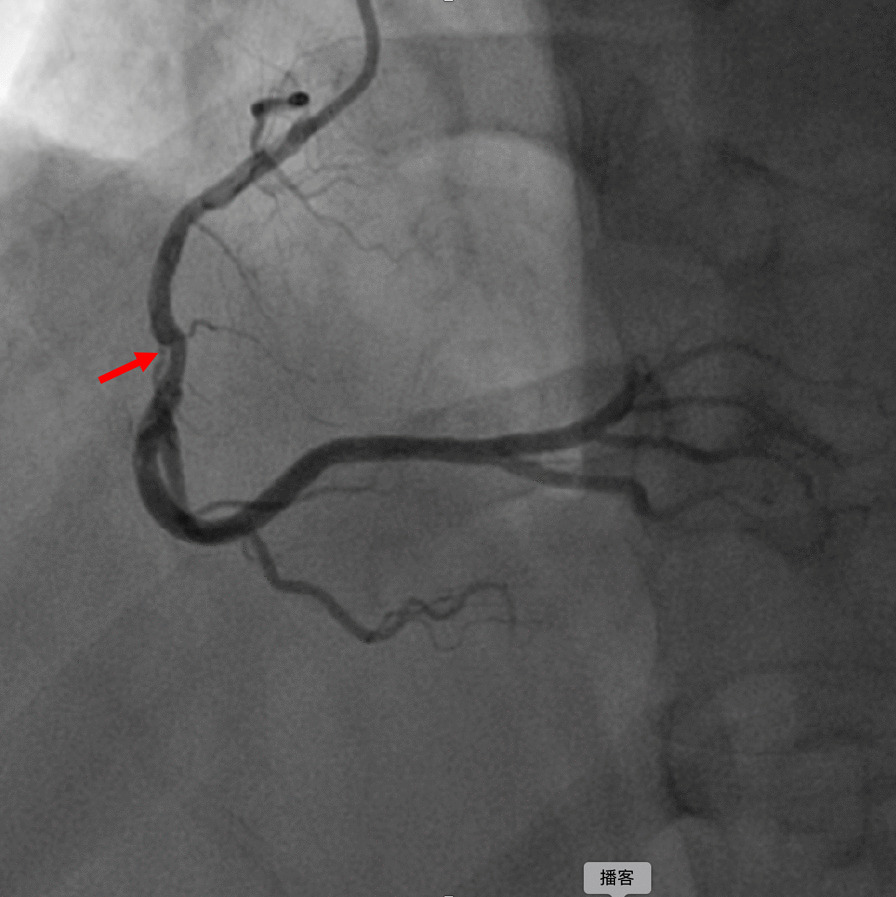
Myocardial bridging (MB) in the middle of RCA with 70% Systolic stenosis (arrow)

During hospitalization, the electrocardiogram (ECG) suggested a sinus rhythm(Fig. 1). TTE showed an apical space-occupying lesion of about 2*1.5 cm and mild tricuspid regurgitation with a normal range of ejection fraction (EF) at 63%. Coronary angiography (CAG) revealed 30% stenosis in both the middle of the left anterior descending branch and the distal left circumflex branch, and 70% systolic stenosis in the middle of the right coronary artery (RCA) along with myocardial bridging (MB) (Fig. 2).

**Fig. 3:**
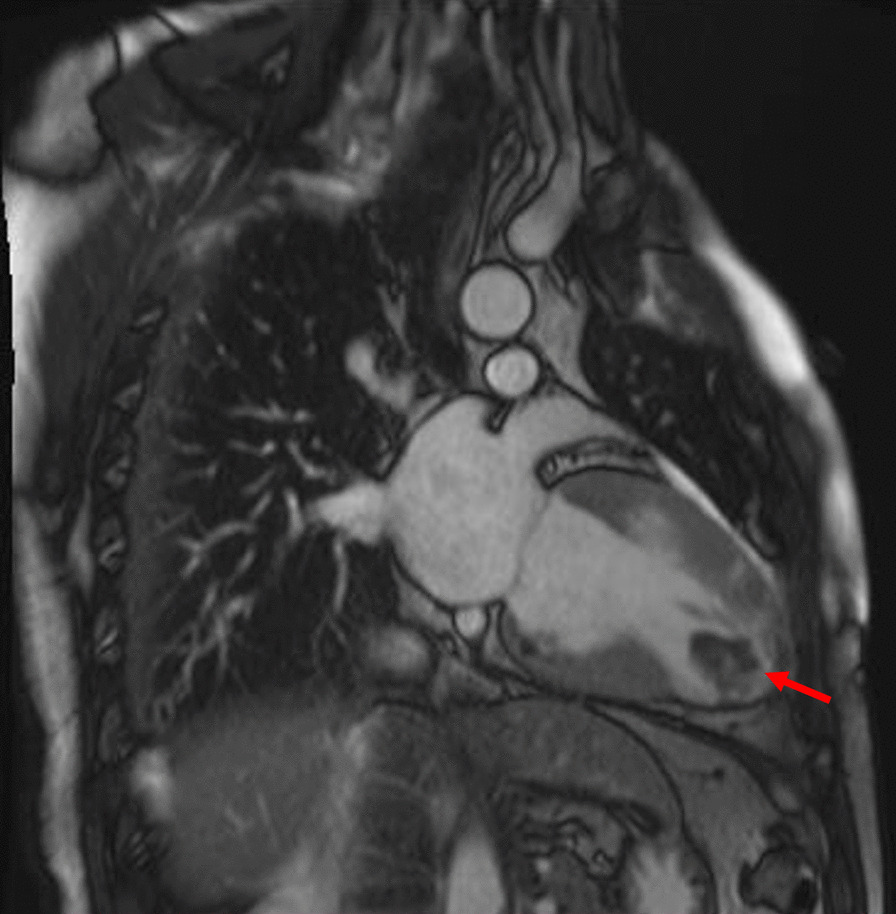
Presence of LV thrombus from magnetic resonance imaging study (arrow)

Besides, Tc-99m myocardial perfusion scintigraphy (resting and activity states) demonstrated that severe myocardial hypoperfusion and hypokinesis in the apex of the left ventricular (LV) were detected. Additionally, the abnormal perfusion myocardium accounted for 19% of the total left ventricular myocardium, with 4% of it being in a hibernated state. Cardiac magnetic resonance imaging (MRI) with delayed enhancement confirmed the formation of the left ventricular aneurysm after myocardial infarction, along with the presence of an apical thrombus (Fig. 3).

**Fig. 4:**
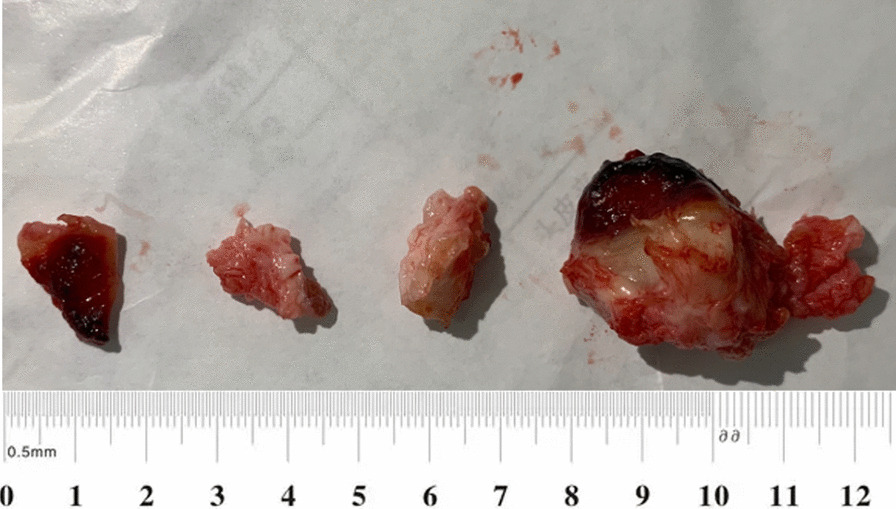
The thrombus was removed from the heart with a size of 3 × 3 cm

**Fig. 5:**
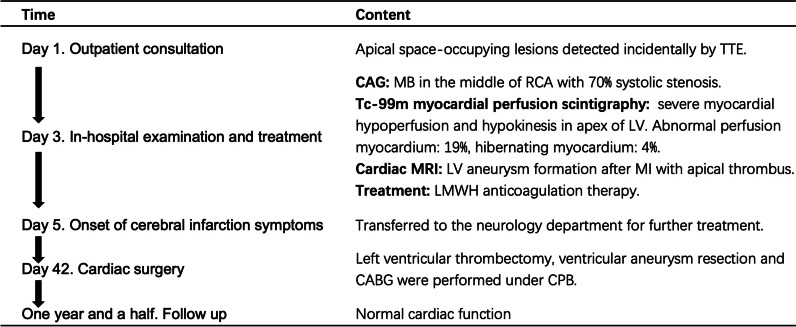
Timeline and major findings during the patient's visit

Two days after the low molecular weight heparin anticoagulant treatment (Nadroparin calcium, 0.4 ml, subcutaneous injection, every 12 h), the patient developed symptoms consistent with cerebral infarction. MRI examination revealed an acute embolism in the left temporal region. Subsequently, the patient was transferred to the neurology department for further treatment. One month later, after ruling out the possibility of acute cerebral infarction by MRI, cardiac surgery was scheduled for the patient. Median sternotomy, cardiopulmonary bypass with moderate hypothermia, and anterograde and retrograde cold blood cardioplegia were performed. With a 3 cm apical incision, the left ventricular thrombus was fully exposed and completely excised (Fig. 4). Thrombotic debris tissue was carefully cleaned after the thrombectomy. The margin of the aneurysm was carefully inspected, and an intraventricular fresh autologous pericardium patch was utilized to reconstruct the left ventricular morphology. Subsequently, the patch was covered by the aneurysm wall, and the wall itself was closed using continuous sutures with two long gaskets. Finally, aorta-to-right coronary artery bypass grafting was performed through the great saphenous vein. The tracheal tube was removed on the first postoperative day. Seven days later, the patient was discharged uneventfully and prescribed warfarin and aspirin therapy for 1 year. The timeline and major findings are summarized in Fig. 5.

## Discussion and conclusions

LVT is mostly found in myocardial infarction, low ejection fraction, LV aneurysms, and ventricular wall akinesis or dyskinesis [4, 5]. The thrombus formation refers to Virchow's triad: blood stasis, endothelial injury, and hypercoagulability [6]. McCarthy et al. reported that the incidence of LVT detected by TTE is only 0.1% in unselected patients. Most LV thrombi are formed within 2 weeks. However, some occur even more later, especially in patients with LV systolic dysfunction [6]. In this case, the patient was unaware of the timing of the left ventricular thrombus formation. It was incidentally discovered during preoperative examination. Echocardiography revealed that the left ventricular thrombus was not a fresh thrombus, indicating that it had been present for a considerable duration of time.

Post-myocardial infarction has been demonstrated as one of the most common risk factors for the development of LVT along with heart failure, alcohol abuse, and tobacco use [1, 7]. In this case, it is worth noting that the patient exhibited three high-risk characteristics including MB in RCA, alcohol abuse, and tobacco abuse, which may be contributed to the development of myocardial infarction. The patient has a history of drinking and smoking for over 10 years, and approximately two years ago, he was transported to the emergency room due to alcohol intoxication. However, the patient has not quit the habit of alcohol abuse and continues to drink excessively on a regular basis. Unfortunately, there has been no improvement in his unhealthy lifestyle. Montone RA et al. reported that coronary spasm with MB is the independent risk factor of myocardial infarction and non-obstructive coronary arteries [8]. During this hospitalization, CAG showed a myocardial bridge in the middle of RCA with 70% systolic stenosis. MRI and myocardial perfusion scintigraphy demonstrated a left ventricular aneurysm with apical thrombus.

Essential thrombocytopenia (ET) is another important incentive that should be considered since it has been demonstrated to attribute to the onset of acute myocardial infarction [9]. To determine if the patient had the ET, complete blood count and gene mutation related to EB were examined. The results showed that the platelet count was 435 × 10^9^ L^−1^ and there was no mutation in JAK2 V617F, JAK2 exon 12, MPL, and CALR in this patient, which has ruled out the possibility of ET.

LVT remains to be a severe complication associated with a high risk of systemic embolism. According to the latest guidelines [10, 11], several anticoagulation therapies are introduced. The current standard therapy for LVT is chronic warfarin therapy for 3 months at the minimum. In addition, direct oral anticoagulants (DOACs) are recently introduced [3, 12]. The therapeutic dilemmas are: which one is the best? How long the treatment course should take? What's the dose? Several studies have suggested that even following strict anti-coagulant treatment, the prognosis of patients is not satisfactory [13, 14]. Although Lattuca et al. demonstrated that prolonged anticoagulation therapy duration could reduce the occurrence of major adverse cardiovascular events, the bleeding complications were raised [13].

Surgical treatment is another considerable option for patients with LVT, especially for those with giant or hypermobile LVT or recurrent systemic emboli developed undergoing anticoagulant therapy [2]. Lee et al. reported that the operative treatment group tended to have less post-treatment thromboembolism than the anticoagulation and antiplatelet groups [15].

Young patients with MB who have longstanding alcohol abuse and smoking habits have a high chance of sudden MI. Although surgical intervention has some inevitable intrinsic risks, patients would benefit from it. Therefore, we highlight that for patients with giant or hypermobile LVT or recurrent systemic emboli, surgical treatment can be a priority of choice.

## Data Availability

All data generated or analyzed during this study are included in this published article.

## Abbreviations

CABG: Coronary artery bypass grafting
CAG: Coronary angiography
ECG: Electrocardiogram
ET: Essential thrombocytopenia
LAD: Left anterior descending branch
LVT: Left ventricular thrombus
MB: Myocardial bridging
MRI: Magnetic resonance imaging
RCA: Right coronary artery
TTE: Transthoracic echocardiography
VKA: Vitamin K antagonist
